# High-Intensity Multimodal Training for Young People: It's Time to Think Inside the Box!

**DOI:** 10.3389/fphys.2021.723486

**Published:** 2021-08-12

**Authors:** Paulo Gentil, Claudio Andre Barbosa de Lira, Rodrigo Luiz Vancini, Rodrigo Ramirez-Campillo, Daniel Souza

**Affiliations:** ^1^College of Physical Education and Dance, Federal University of Goiás, Goiânia, Brazil; ^2^College of Physical Education and Sport, Federal University of Espírito Santo, Vitoria, Brazil; ^3^Department of Physical Activity Sciences, Universidad de Los Lagos, Santiago, Chile; ^4^Centro de Investigación en Fisiología del Ejercicio, Facultad de Ciencias, Universidad Mayor, Santiago, Chile

**Keywords:** sedentary behavior, pediatric obesity, resistance training, high-intensity interval training, plyometric exercise, human physical conditioning, physical exercise, interval training

## Introduction

Studies comparing children and adolescents from different periods have shown a decrease in physical activity and fitness in the last decades (Moliner-Urdiales et al., 2010; Runhaar et al., 2010; Cohen et al., 2011; Hardy et al., 2013; Santtila et al., 2018; Masanovic et al., 2020; Fühner et al., 2021). Low physical fitness is associated with poor metabolic health, independent of central adiposity (Lätt et al., 2018). Moreover, changes in physical fitness from childhood and adolescence to adulthood are related to metabolic health, physical activity levels, and bone mineral density (García-Hermoso et al., 2019; Mäestu et al., 2020), while both low cardiorespiratory fitness and muscle strength are associated with higher risk of premature death and disability (Ortega et al., 2012; Henriksson et al., 2019a,b). Obesity is another important concern (Wijnhoven et al., 2014; Ogden et al., 2016; Bentham et al., 2017), but previous studies have shown that increased physical activity might be associated with decreased adiposity (Hui et al., 2021) and can modulate the effects of genetic predisposition to obesity in young people (Todendi et al., 2021).

The negative impact of a sedentary lifestyle during early life has been largely debated (Faigenbaum et al., 2011; Faigenbaum and Myer, 2012; Stracciolini et al., 2013). In the early 60s, Kraus and Raab (1961) stressed the importance of physical activity for preventing diseases and suggested that the adverse health effect of physical inactivity was comparable to lack of vitamins or contagious diseases. Based on this, Faigenbaum et al. (2013) proposed a population-wide approach for identifying inactive children, prescribing interventions and raising public awareness.

Notwithstanding, some important barriers for physical activity adoption among children and adolescents might be considered, like perception of lack of safety, physical environment and lack of support (Stankov et al., 2012; Lu et al., 2014; Martins et al., 2014). It is also important to consider children and adolescents particularities (e.g., biological and behavior characteristics) when prescribing and evaluating exercise programs, since exercise programs designed for adults might be inadequate for them (Faigenbaum et al., 2013).

Previous studies suggested that young people naturally engage in intermittent activities (Bailey et al., 1995) and high intensity activities might be particularly beneficial to improve cardiorespiratory fitness (Baquet et al., 2003; Costigan et al., 2015), mental health (Leahy et al., 2020), body composition (Costigan et al., 2015; D et al., 2019) cardiovascular risk and metabolic health (Cooper et al., 2016; García-Hermoso et al., 2016; MA et al., 2021) in children and adolescents.

The purpose of the current article is to present the benefits of high-intensity multimodal training (HIMT) programs, such as CrossFit, to the youth, with a critical discussion about its potential benefits and concerns.

## Potential Benefits

HIMT involves exercise programs that mix many different exercise modalities (e.g., weightlifting, powerlifting, gymnastic, calisthenic, plyometrics, running, and others) and train multiple physical capacities at the same time (e.g., cardiorespiratory, muscle strength, and flexibility) (Feito et al., 2019). The performance of high-intensity exercises in an intermittent and station-based fashion, might confer to HIMT characteristics similar from existing training methods such as circuit training and high-intensity interval training (Sobrero et al., 2017; Feito et al., 2019). Probably, the most popular form of HIMT is CrossFit; however, there are many other activities and names that might be included in this definition, like high-intensity functional training, cross-training and others.

HIMT programs are usually designed for improving physical fitness and motor skills, being characterized by high levels of effort, with a great stress in cardiorespiratory and neuromuscular systems (Timón et al., 2019). These activities have been reported to promote marked increases in muscle mass and strength and cardiorespiratory fitness, and reduce body fat (Murawska-Cialowicz et al., 2015; Brisebois et al., 2018; Carnes and Mahoney, 2019; Bahremand et al., 2020), which are, in some cases, higher than conventional activities (Bahremand et al., 2020). There is evidence that HIMT participants present high levels of satisfaction and motivation (Claudino et al., 2018) and perceive it as more enjoyable than conventional training (Heinrich et al., 2014).

CrossFit Teen and CrossFit Kids are similar programs adapted from CrossFit and designed specifically to improve fitness and resistance skill of young population. In agreement with the studies involving adults, such HIMT programs have shown to improve physical fitness in children and adolescents (Eather et al., 2016b; Garst et al., 2020), with high rates of attendance (94%) and satisfaction (4.2–4.6 out of 5) (Eather et al., 2016b). HIMT like CrossFit is usually performed inside a specific facility named “Box” (Feito et al., 2019); however, programs adapted for young people involves minimal equipment and can also be easily implemented at school setting (Eather et al., 2016b; Garst et al., 2020). In this context, the effects of HIMT on health-related fitness of students are comparable to those obtained with the regular participation in physical education classes (Garst et al., 2020).

The higher intensity achieved during HIMT seems to be an important aspect for cardiorespiratory fitness in the youth, since exercising at intensities above 80% of maximal heart rate might be important for this group (Baquet et al., 2003). Previous studies have suggested that young people might particularly benefit from high-intensity physical exercise, with improvements in body composition, metabolic, and cardiovascular health (Gist et al., 2014; Cooper et al., 2016; Eddolls et al., 2017; García-Hermoso et al., 2020), but also in other components like cognitive and mental health (Leahy et al., 2020). Regarding the latter, 8 weeks of HIMT intervention brings mental health benefits among adolescents at increased risk for psychological stress (Eather et al., 2016a). Thus, HIMT can be considered a helpful strategy to manage mental health issue in school-aged individuals.

The muscle strengthening component of HIMT might also be important for the youth, since muscle strength seems to has a strong association with health benefits (García-Hermoso et al., 2019) and mitigate the worsening of metabolic health associated with insufficient levels of physical activity (Gomes et al., 2017). Children and adolescents have lower levels of sexual hormones compared to adults, and also reduced strength and power when normalized by body mass (Dotan et al., 2012; Dotan, 2016). However, besides these apparent limitations, there are many studies showing that children and adolescents are capable of increasing muscle strength and mass in response to training (Faigenbaum et al., 1996; Pikosky et al., 2002; Granacher et al., 2011; Assunção et al., 2016). Of note, some of these studies involve children as young as 5 years old (Faigenbaum et al., 1999).

According to Faigenbaum et al. (2016), education and instruction on proper resistance training techniques and procedures should start early in life. Neuromuscular performance and muscle strength might positively predict motor competence in children (Wright et al., 2020) and higher motor competence during childhood is associated with sustained physical activity practice in adolescence (Larsen et al., 2015).

## Potential Concerns

Injury risk is probably the main concern regarding HIMT in adults (Claudino et al., 2018). However, data reporting the prevalence or incidence of injury in young HIMT practitioners is scarce. A retrospective study about pediatric CrossFit-related injury found that the absolute number of CrossFit-related injury in young increased over time since CrossFit foundation (Stracciolini et al., 2020). This crescent CrossFit-related injury rate could be associated with the increased participation of young people in this modality, while the absence of relative risk analyses makes difficult to classify or compare injure rate with another training modality. The profile of CrossFit-related injury in the young might differ between sex and age, with higher proportion of lower limb injury in women, higher proportion of Shoulder injury in men, and higher proportion of trunk/spine for participant younger than 20 years old (Sugimoto et al., 2020). These findings might be of particular interest in order to develop safe HIMT for young practitioners.

Other possible concerns involving HIMT for adults, like insufficient recovery between exercises and sessions, concurrent effects and lack of specificity, might not be harmful or can even be advantageous when considering young people.

It has been suggested that recovery from high-intensity exercises (i.e., all out sprints and Wingate tests) is faster in children and adolescents when compared to adults (Bar-Or, 1995; Ratel et al., 2004). Moreover, children and adolescents seem to naturally engage in intermittent activities (Bailey et al., 1995) and studies have reported that they usually consider it enjoyable (Ratel et al., 2004; Malik et al., 2017, 2018).

The faster recovery in the young has also been reported in resistance training. Faigenbaum et al. (2008) compared the performance in the bench press exercise at 10 repetitions maximum (10RM) load between children, adolescents and adults. The results showed that children recovered faster than adolescents and adults, while adolescents recovered faster than adults. Therefore, using short interval lengths might not interfere with the results in younger people (Ramirez-Campillo et al., 2014, 2019b; Drury et al., 2021).

Regarding the recovery between sessions, a previous study by Soares et al. (1996) compared the recovery of children (12 years old) and adults (28 years old) after 5 sets of bench press performed to momentary muscle failure. According to the results, there were no changes in indirect markers of muscle damage [isometric strength and creatine kinase (CK) levels] 24 h after training in children, while adults did not recover for as long as 72 h. Similar findings were reported by Chen et al. (2014), that used muscle damaging protocols (5 sets of 6 maximal eccentric elbow flexions) and found that recovery was faster in children than adolescents and adults; and in adolescents when compared to adults. Later, Deli et al. (2017) compared the responses of boys (10–12 years old) and adults (18–45 years old) to maximal eccentric knee extensions and confirmed that children are less susceptible to exercise-induced muscle damage than adults. Children and adolescents seem to require less days to recover from a resistance exercise session than adults (Ramírez-Campillo et al., 2015) and a higher training frequency promotes higher increases in muscle strength in this group (Moran et al., 2017). Therefore, the concerns regarding insufficient recovery during HIMT might not be a problem for the young.

HIMT also can involves a large component of plyometric jumps, which might improve physical performance in young people and are safe over short term (De Freitas Guina Fachina et al., 2017; Assunção et al., 2018; Ramirez-Campillo et al., 2019a,b; Vera-Assaoka et al., 2020). However, it is important to remember that overuse injuries and tendinopathies are frequent in young athletes (Le Gall et al., 2006; Johnson et al., 2020), which might be due to an imbalance between muscle and tendon adaptation (Mersmann et al., 2014, 2016, 2017). Considering that resistance training might increase tendon strength (Kongsgaard et al., 2007; Martins et al., 2018), it is recommended to design programs with an adequate balance between plyometric (particularly high-impact jumps) and resistance training volumes, specially adolescents. The combination of resistance and plyometric training might also be beneficial for increasing performance (Zghal et al., 2019; Thapa et al., 2021). Radnor et al. (2017) studied the response of children of different maturity groups (pre- or post-peak height velocity) to a plyometric training, resistance training and combined training and reported that, irrespective of maturation, combined training provided the greatest improvements in performance.

The combination of resistance and aerobic training during HIMT might be a concern because of the potential concurrent effects. However, a systematic review with meta-analysis reported that concurrent training was more effective than single mode aerobic or resistance training in improving physical fitness in children and adolescents (Gäbler et al., 2018). Interestingly, the study revealed that concurrent training promoted higher increases on muscle power in young people when compared to strength training alone, which is the opposite to what have been reported in adults (Wilson et al., 2012).

There might be some concerns with the strengthening exercises used during HIMT, because this might be associated with risk of injury in children. However, the injury risk for these activities are lower than other sports, like soccer and basketball (Hamill, 1994), being considered safe for the youth (Falk and Eliakim, 2003; Malina, 2006; Faigenbaum et al., 2009).

Myer et al. (2009) evaluated injuries seen during emergency room visits associated with resistance training (weightlifting). The results revealed that accidental injuries decreased with age, while sprain/strain injuries increased. More than two thirds of the injuries sustained in the 8–13 group occurred in the heads, hands and feet and were most often related to “dropping” and “pinching”. Therefore, it seems that children have lower risk of sprains and strains, but a higher risk of accidental injuries, suggesting the need for adequate (and qualified) supervision during training sessions.

Because of the highly fatiguing nature of HIMT, proprioception and exercise technique are likely altered, compromising safety and efficacy of such programs, particularly for those involving exercise that require complex technics such Olympic-style weightlift (e.g., snatches and clean, and jerks), as suggested by Hooper et al. (2014). Therefore, children and adolescents must be closely supervised during HIMT and, if necessary, training programs should be adapted for their individual characteristics.

The highly fatiguing nature of HIMT should also be considered when training close to academic tasks. Though HIMT might be beneficial to improve motor skills it can impair academic performance in middle school students, due to an impairment in concentration capacity (Garst et al., 2020). However, this seems to be controversial since other studies showed that high intensity training improves executive function, memory, and selective attention in children and adolescents (Ma et al., 2015; Moreau et al., 2017; Lind et al., 2019; Tottori et al., 2019). Therefore, the negative results found by (Garst et al., 2020) might be specific to the modality used (CrossFit) and not due to high intensity nature of the activity *per se*. There is a common belief that high effort, especially lifting weight might limit longitudinal growth in children and adolescent. However, exercise might positively influence longitudinal growth (Borer, 1995; Hills and Byrne, 2010) and there is no evidence that muscle strengthening activities have a negative impact on growth (Falk and Eliakim, 2003; Malina, 2006; Faigenbaum et al., 2009). In fact, HIMT might be even beneficial, since high intensity activities increase circulating human growth hormone in children and adolescents (Saggese et al., 1987; Eliakim and Nemet, 2008, 2013).

## Final Comments

Although there is no minimum age to start exercising (Myer et al., 2011; Faigenbaum et al., 2016), it is necessary to adapt training programs to the youngsters' biological and behavioral characteristics. Children and adolescents should be able to understand and follow instructions and they should receive safety instructions on lifting weights, proper spotting and equipment use.

Considering the competitive nature of some HIMT programs, it is important to remember that untrained youth tend to overestimate their physical performance, which might increase the risk of injury (Plumert and Schwebel, 1997). This highlights the importance of qualified supervision, which is further reinforced by the fact that direct supervision improves program adherence and the results in young people (Coutts et al., 2004).

Professionals involved with HIMT for children and adolescents need to acknowledge both their biological characteristics and psychological uniqueness. Professionals should be particularly sensitive to children and adolescents who are overweight/obese and with low physical capacities. Although they are the ones who potentially will get most benefit from HIMT, they are also the ones who might be more reluctant to adhere.

Some practical suggestions to improve adherence of obese pediatric population using HIMT, should be access to a gym, initial direction by a trainer, variety, and group-based activities (Peeters et al., 2012). Other important factor are the support of family and peer (Peeters et al., 2012; Salvy et al., 2012; Sundar et al., 2018); therefore, it is important to involve them in the exercise programs as much as possible. It is also important to provide constant feedback regarding improved fitness, since obese children might join exercise initially aiming at losing weight, but focused more on fitness over time (Peeters et al., 2012). Considering that mastering the activity is associated with less motivation (Sundar et al., 2018), we suggest choosing simple exercises and progress carefully.

**Figure 1:**
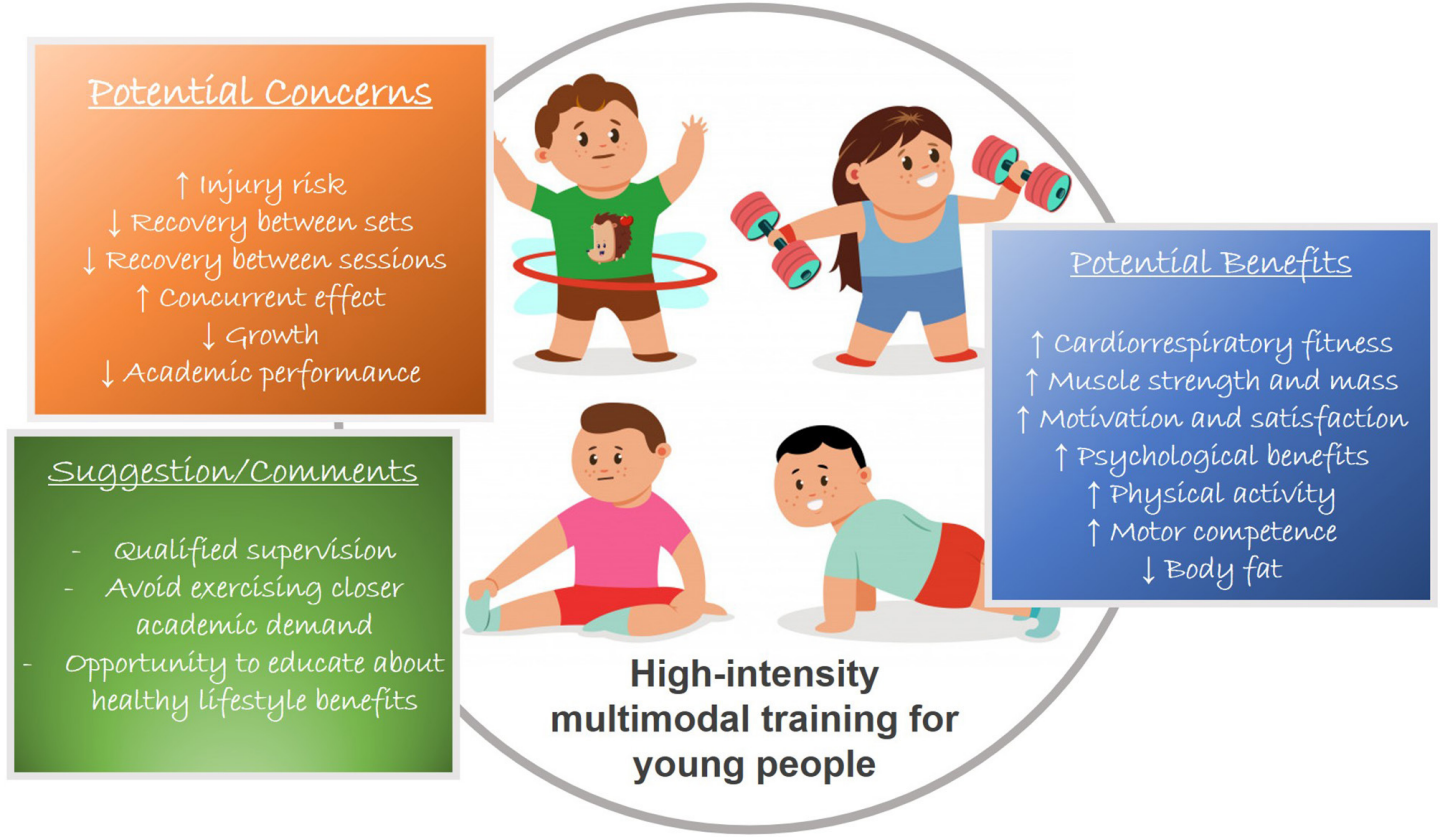
Summary of benefits (Eather et al., 2016a,b), concerns (Hooper et al., 2014; Claudino et al., 2018; Garst et al., 2020), and suggestion/comments (Myer et al., 2011; Faigenbaum et al., 2016), regarding high-intensity multimodal training in young people.

In technical terms, the characteristics of HIMT, such as, the simultaneous development of many physical capacities and movements and exercise diversity might be particularly interesting for training young people. HIMT involves some important aspects for exercise adherence like variety and group-based, and might easily involve others like access to exercise facilities and supervision (Peeters et al., 2012). Many concerns like an increased risk of injury, insufficient recovery might not be troublesome for this group and are not difficult to address (Figure 1).

During HIMT exercise professionals might have an opportunity to promote positive changes in physical function and body composition in children and adolescents, as well as to promote improvements in mental health and psychosocial aspects. Moreover, this might be an important opportunity to educate them about the benefits of a healthy lifestyle and overcome the perceived barriers to being physically active. The increase in physical fitness might increase spontaneous participation in physical activity and sports (Eiholzer et al., 2010; Fransen et al., 2014). Therefore, HIMT might be seen as an end (to increase physical fitness) but also as a mean (to increase physical activity).

## Author Contributions

PG, CABdL, RLV, RR-C, and DS: conception, drafting the article, revising it critically, and final approval of the version to be published. All authors contributed to the article and approved the submitted version.

## Conflict of Interest

The authors declare that the research was conducted in the absence of any commercial or financial relationships that could be construed as a potential conflict of interest.

## Publisher's Note

All claims expressed in this article are solely those of the authors and do not necessarily represent those of their affiliated organizations, or those of the publisher, the editors and the reviewers. Any product that may be evaluated in this article, or claim that may be made by its manufacturer, is not guaranteed or endorsed by the publisher.
